# Mortality in Cuba, and the Necessity for Its Acquisition by the United States

**Published:** 1898-05

**Authors:** 


### MORTALITY IN CUBA, AND THE NECESSITY FOR ITS ACQUISITION BY THE UNITED STATES.

On account of the declarations contained in the war resolutions adopted by Congress at the beginning of the war with Spain, the government of the United States is so handicapped as to preclude the possibility of acquiring title or exclusive sovereignty over the island Cuba, and yet leaving out of consideration the immense commercial advantages, and the great value of the island from a strategic standpoint, as a key to the Mexican gulf, and viewing the matter only as a sanitary measure, the importance to the people of our Southern cities, of possession of Cuba by the United States, can scarcely be overestimated.

Under the government of Spain there is little or no prospect of improvement in the sanitary condition of the island, nor can such improvement be reasonably expected under a government by the Cubans themselves. In order that our readers may form some idea of the dangers from infectious and pestilential diseases that are perpetually threatening us at our very doors, and of the wretched sanitary condition of the island, it is only necessary to glance at some reports of the Marine Hospital Service. Under date of April 25th, 1898, Dr. W. F. Brunner makes a report to the Acting Surgeon General of the greatest interest and importance.

The report is entitled, "Morbidity and Mortality in the Spanish Army in Cuba During the Calendar Year 1897."

"The following mortality from yellow fever in the five military hospitals in Havana is correct, it being taken from official figures and verified by careful investigation."

| MONTHS. | HAVANA. | REGLA. | TOTAL. |
|---|---|---|---|
| January | 152 | 109 | 261 |
| February | 43 | 74 | 117 |
| March | 42 | 56 | 98 |
| April | 76 | 112 | 188 |
| May | 89 | 102 | 191 |
| June | 181 | 234 | 415 |
| July | 211 | 237 | 438 |
| August | 185 | 112 | 297 |
| September | 179 | 138 | 317 |
| October | 71 | 57 | 128 |
| November | 48 | 53 | 101 |
| December | 17 | 15 | 32 |
| Total | | | 2583 |

This mortality represents, according to the report, about 10,000 cases of yellow fever, which shows a mortality of a little more than 25 per cent. of the total number of cases. Further on the report gives the number of deaths from yellow fever in the following named cities during the year 1897:

| | |
|---|---|
| Mantanzas | 238 |
| Santiago de Cuba | 658 |
| Sagua la Grande | 378 |
| Cardenas | 235 |
| Cienfuegas | 212 |
| Holguin, Guimas, Remedios, Sancti Spiritis, etc | 1500 |
| Total | 3221 |
| Deaths in Havana | 2583 |
| Total deaths from yellow fever in military hospitals, 1897 | 5804 |

This total mortality represents about 30,000 cases.

The Spanish army did not suffer greatly from small-pox during the year, but the civil population of every town in Cuba was decimated by the ravages of the disease. In the city of Havana alone there were 1296 deaths from small-pox, and as great numbers of the houses are infected, it would be exceedingly imprudent for an army of the United States to occupy the city unless previous vaccination was universal. But the tale of horror is not yet finished. It is estimated that the army of Spain lost during the year 1897:

| | |
|---|---|
| From Enteric fever | 2,500 |
| Malarial fevers | 7,000 |
| Enteritis and dysentery | 12,000 |
| All other diseases | 5,000 |
| Add deaths from yellow fever | 5,804 |
| Total deaths from all diseases | 31,300 |

These figures only represent the mortality from diseases in Spain's army of occupation for one year. Out of a total of approximately 100,000 men about one-third have died of disease in one year.

According to this ratio of deaths it would require about two more years to finish the work of destruction of the Spanish army without help from either the Cubans or the United States.

But these mortality figures in the army, horrible as it may appear, we think by no means represent the worst conditions existing on the island. It must be remembered that these mortality statistics were gathered from the best fed, clothed and cared-for class in Cuba. Assuming this to be true, what must have been the condition of the houseless, the homeless and the starving? Accurate reports from this unhappy class will probably never be obtained, but we are not wholly without information. Intelligent American citizens who have recently visited Cuba and carefully observed the condition of the so-called reconcentradoes, have declared that their desperate and hopeless condition has no parallel in modern times.

It is needless to attempt to portray the fearful sanitary conditions surrounding these people, as it has already been repeatedly described in the newspapers of this country. Enough is known to warrant the belief that during the past three years at least one-fourth of the entire population of the island have perished from diseases resulting from insanitary conditions, and starvation. Even in the city of Havana, the metropolis of the island, under military rule, there is, apparently, almost a total disregard of all sanitary laws.

The disease known as glanders, in this country confined to horses, is, according to Dr. Brunner's report, quite common in the Spanish army, and glandered horses are seen daily on the streets of Havana. Leprosy is also common, and hundreds of this unhappy class wander about the city at will. Surely it should not be matter for surprise that such barbarous conditions have touched the deepest sympathies of a great and enlightened people, and that they, through their government, have resolved to expel the Spaniard and bring peace, order, liberty and happiness to what remains of the people of Cuba. The only difficulty in the way of complete success is the before-mentioned resolutions, by which the government has committed itself to turn over the government of Cuba to the people thereof as soon as the Spanish are expelled.

